# Onset of deaminase APOBEC3B induction in response to DNA double-strand breaks

**DOI:** 10.1016/j.bbrep.2018.10.010

**Published:** 2018-10-30

**Authors:** Atsuhiro Shimizu, Haruka Fujimori, Yusuke Minakawa, Yusuke Matsuno, Mai Hyodo, Yasufumi Murakami, Ken-ichi Yoshioka

**Affiliations:** a Division of Carcinogenesis and Cancer Prevention, National Cancer Center Research Institute, 5-1-1 Tsukiji, Chuo-ku, Tokyo 104-0045, Japan; b Department of Biosciences, School of Science, Kitasato University, 1-15-1 Kitasato, Minami-ku, Sagamihara 252-0373, Japan; c Biological Science and Technology, Tokyo University of Science, 6-1-1 Niijuku, Katsushika-ku, Tokyo 125-8585, Japan; d Department of Applied Chemistry, Faculty of Science, Tokyo University of Science, 1-3 Kagurazaka, Shinjuku-ku, Tokyo 162-8601, Japan

**Keywords:** BER, base excision repair, DSB, double-strand break, UNG, uracil-DNA glycosylase, ATM, ataxia-telangiectasia mutated, ATR, ataxia-telangiectasia and Rad3-related, APOBEC3B, Deaminase, Genomic instability, Base excision repair, Ataxia-telangiectasia and Rad3-related, Uracil-DNA glycosylase

## Abstract

Deamination of 5-methyl cytosine is a major cause of cancer-driver mutations in inflammation-associated cancers. The deaminase APOBEC3B is expressed in these cancers and causes mutations under replication stress; however, the mechanisms by which APOBEC3B mediates deamination and its association with genomic disorders are still unclear. Here, we show that APOBEC3B is stabilized to induce deamination reaction in response to DNA double-strand breaks (DSBs), resulting in the formation of long-lasting DSBs. Uracil, the major deamination product, is subsequently targeted by base excision repair (BER) through uracil-DNA glycosylase 2 (UNG2); hence late-onset DSBs arise as by-products of BER. The frequency of these delayed DSBs was increased by treatment of cells with a PARP inhibitor, and was suppressed following knock-down of UNG2. The late-onset DSBs were induced in an ATR-dependent manner. Those secondary DSBs were persistent, unlike DSBs directly caused by γ-ray irradiation. Overall, these results suggest that the deaminase APOBEC3B is induced in response to DSBs, leading to long-lasting DSB formation in addition to mutagenic 5me-C>T transition induction.

## Highlights

• APOBEC3B is stabilized to induce deamination reaction in response to DSBs.
• Deamination leads to formation of late-onset DSBs through UNG2-mediated BER.
• Late-onset DSBs induced in an ATR-dependent manner are persistent.

## 1 Introduction

Cancer development is associated with mutations and genomic instability [1], [2]. In cancers that develop under conditions of chronic inflammation, deamination-associated C>T mutations are induced massively in association with expression of deaminase APOBEC3A and B [3]. These C>T mutations occur widely in epigenetically-methylated CpG islands; this is because deamination of 5me-cytosine yields thymine (5me-C>T transition) [4]. While APOBEC3A is more effective in 5me-C deamination than APOBEC3B, deamination mediated C>U induction by APOBEC3A is less efficient than that by APOBEC3B [5], [6]. APOBEC3A and B targets single strand DNA (ssDNA); therefore, it plays a crucial role in restricting proliferation of retroviruses by hyperediting their complementary DNA intermediates [7]. Deamination may also occur at exposed ssDNA sites in normal cells. While deamination at unpaired ssDNA loci could result in G/T-mismatches that are repairable by base excision repair (BER) (via the thymine DNA glycosylases TDG and MBD4) [8], [9], deamination at exposed ssDNA sites could lead to mutational 5me-C>T transitions in addition to major C>U transitions that are repairable through UNG-mediated BER [10]. Indeed, deamination-mediated mutagenesis, i.e., 5me-C>T transition, is elevated in APOBEC3B-expressing cells [5] and is a major pathway that results in cancer-driver mutations, such as those present in the *p53* and *APC* genes [11].

Deamination-mediated mutations (5me-C>T transitions) at CpG sites are induced in association with kataegis, a mutational process that results in localized hypermutations at or around genome rearrangement loci [12]. Because genome rearrangement occurs via erroneous end-joining of double-strand breaks (DSBs), deamination-mediated hypermutation may occur during the damage response or repair processes. Intriguingly, deamination-mediated mutagenesis occurs in the lagging strand under replication stress in a manner that is dependent on the ataxia-telangiectasia and Rad3-related (ATR) kinase [13]. However, the association between deamination and damage responses is still not clear. In addition, although deamination is usually induced at very limited genomic loci [12] (where it should also induce massive C>U transitions), it remains unclear whether such massive transitions are associated with genome integrity.

In this study, we found that DSBs trigger the onset of APOBEC3B activation, in a manner dependent on ATR. Subsequently, the cells undergo massive BER via nuclear uracil-DNA glycosylase UNG2, ultimately leading to further DSB accumulation.

## 2 Materials and methods

### 2.1 Cell culture

HeLa and SW480 cells were obtained from ATCC and cultivated in DMEM containing 10% fetal calf serum [14]. The cells were transfected with the APOBEC3B expression vector (pPM-APOBEC3B; abm) or the negative control vector (pPM-NC; abm) using Lipofectamine 3000 (Life Technologies). Treatment with olaparib (Selleckchem), KU55933 (Merck Millipore), VE-822 (Selleckchem), or NU7441 (Selleckchem) was performed where indicated. To knockdown nuclear UNG2, a siRNA that targets both mitochondrial UNG1 and UNG2 (Thermo Fisher; 139934) were used where indicated, comparing with a negative control siRNA (Qiagen; 1027281). Survival rates were determined by counting the number of viable cells 7 days after γ-ray irradiation.

### 2.2 DNA damage

**Fig. 1:**
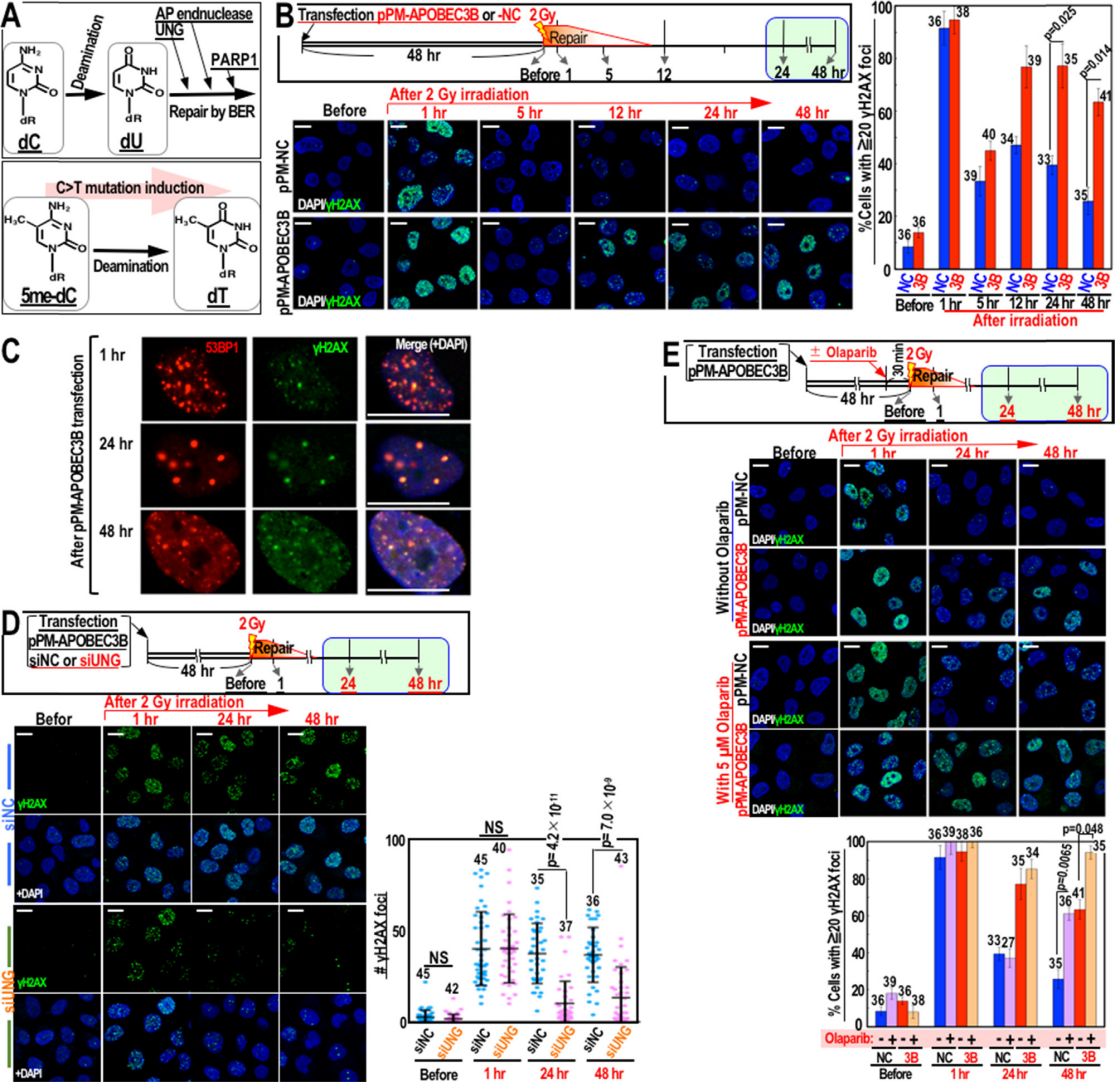
DSBs trigger activation of APOBEC3B and lead to late-onset DSB formation mediated by BER. (A) Schematic representation of deamination reactions and the subsequent BER process. (B–C) HeLa cells were transfected with the pPM-APOBEC3B or negative control (NC) vector and treated as depicted in the upper boxes. The numbers of γH2AX foci in γ-irradiated HeLa cells were quantified via immunofluorescence (B). γH2AX foci merged with 53BP1 foci: 97% at 1 h, 90% at 24 h, and 84% at 48 h (C). (D) Cells were transfected with a negative control siRNA (siNC) or a siRNA against UNG (siUNG). (E) Cells were treated with or without the PARP inhibitor olaparib. Bars show means ± s.d. The numbers of cells counted in each condition are inserted in the graphs. Scale bars, 10 µm.

DNA damage was induced by 137Cs irradiation of cells in a Gammacell 40 Exactor (Best Theratronics). The induced DSBs were detected by immunostaining of γH2AX and 53BP1. Hydroxyurea (Sigma) was also used to induce DNA replication stress-associated DSBs. Statistical analysis of γH2AX foci was performed from the indicated numbers of cells. In Figs. 1B and 1D, statistical analyses were performed together for the experiments done in same condition.

### 2.3 Antibodies, immunostaining, and western blotting

Antibodies against the following proteins were obtained from the indicated suppliers: γH2AX (Millipore, 05–636; and CST, 9718), β-actin (Sigma, AC-74), 53BP1 (Merck, PC712), APOBEC3B (GeneTex, GTX17214), and HA-tag (Abcam, ab49969). Western blotting was performed as described previously [15]. Immunostaining was also performed as described previously [16] using a confocal laser microscope (Olympus, FV10i). Before immunostaining with primary and secondary antibodies, cells were fixed with 4% paraformaldehyde for 10 min and permeabilized with 0.1% Triton X-100/PBS for 10 min. For confocal microscope imaging, cells were cultured on coverslips and stained as above.

## 3 Results

### 3.1 APOBEC3B is activated in response to DSBs

The APOBEC3B deaminase targets both cytosine and 5me-cytosine in ssDNA [17] (Fig. 1A). While deamination of 5me-cytosine is induced only at epigenetically-methylated DNA loci (which leads to 5me-C>T transitions), deamination of cytosine should be induced widely; this causes C>U transitions that subsequently induce UNG2-initiated BER to eliminate uracil from the DNA strands in the nucleus [10]. To determine the deamination status of cellular APOBEC3B in response to DNA damage, DSBs that appeared as by-products of BER were analyzed in irradiated cells expressing an APOBEC3B-containing or negative control vector. Because large numbers of DSBs are induced during BER in association with DNA replication [18], late-onset DSB formation was evaluated 12–48 h after γ-irradiation (2 Gy) by monitoring the formation of γH2AX foci. As expected, after the early repair of DSBs that were directly caused by the γ-irradiation, secondary γH2AX foci were specifically observed in APOBEC3B-expressing cells 12–48 h after γ-irradiation (Fig. 1B). In addition, most γH2AX foci merged with 53BP1 foci (Fig. 1C), indicating that the γH2AX foci were DSBs. Such late-onset γH2AX foci were observed in most of the APOBEC3B-expressing cells, and the level of induction of these foci was almost identical to that caused by the γ-irradiation. In addition, late-onset γH2AX foci were observed continuously throughout the monitoring period, indicating that, unlike the DSBs caused by irradiation directly, they were not efficiently repaired.

To gain further insights into the roles of deamination and BER in late-onset DSB formation, we examined the effects of siRNA-mediated knock-down of UNG2, a nuclear uracil-DNA glycosylase, on delayed γH2AX foci formation (at 24–48 h) in cells overexpressing APOBEC3B. As expected, the delayed formation of γH2AX foci in these cells was effectively suppressed by knock-down of UNG2 (Fig. 1D), suggesting that late-onset DSB formation is dependent on UNG2-mediated BER, thereby supporting the induction of APOBEC3B-mediated C>U transitions. This hypothesis is further supported by the finding that treatment of cells with olaparib, a PARP inhibitor that blocks the ligation step of BER, enhanced the accumulation of late-onset γH2AX foci (Fig. 1E). Taken together, these results support the hypothesis that APOBEC3B is activated by γ-irradiation and leads to C>U transitions, which subsequently result in UNG2-mediated BER and the induction of γH2AX foci as by-products. Unlike the early-onset γH2AX foci directly caused by γ-irradiation, the secondary γH2AX foci were continuously observed throughout the monitoring period, suggesting that the secondary damage caused by deamination and BER was persistent (Fig. 1A).

### 3.2 ATR is required for APOBEC3B-mediated late onset DSB formation

**Fig. 2:**
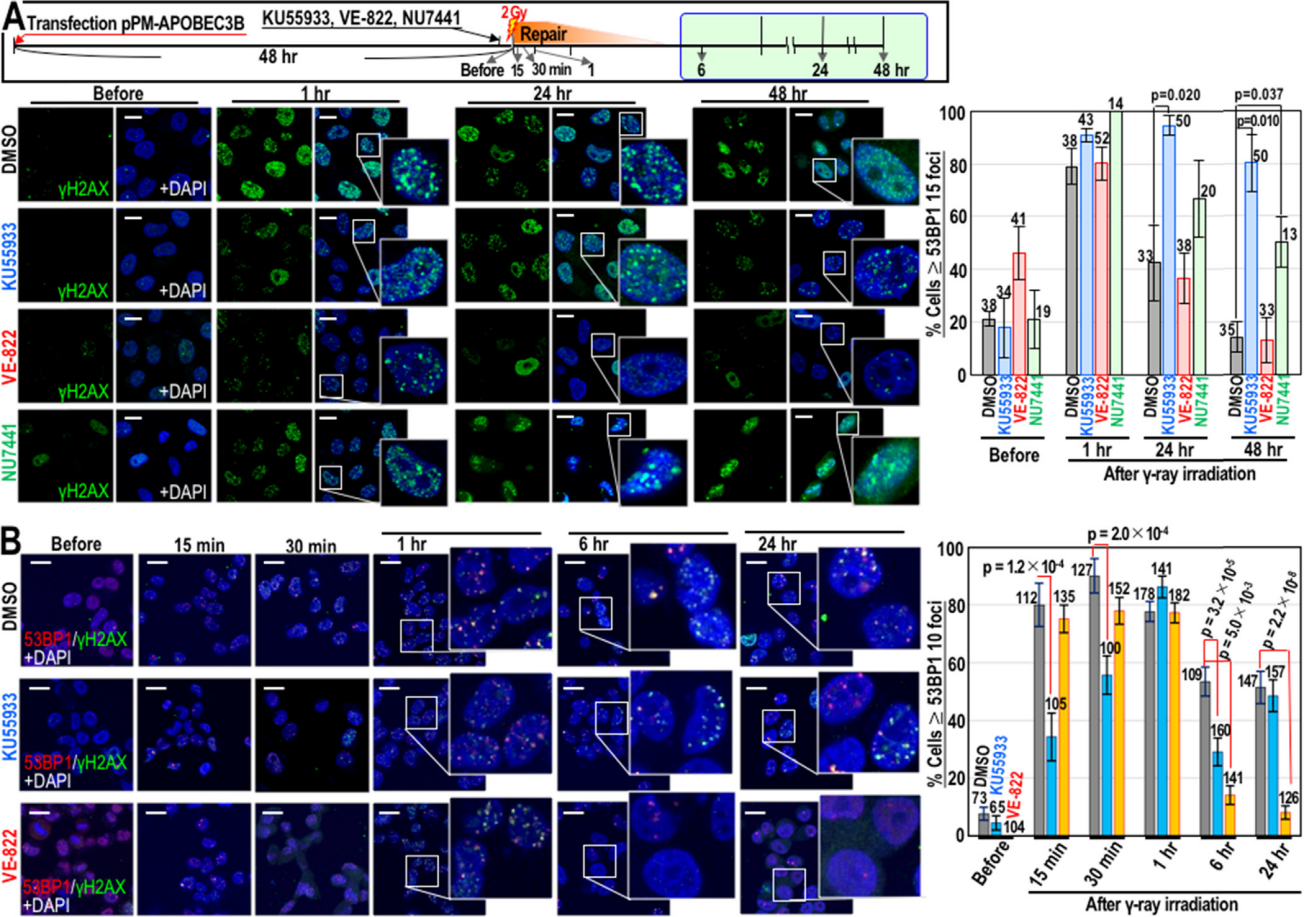
The formation of late-onset DSBs in the presence of APOBEC3B requires ATR. (A, B) HeLa cells were treated as depicted in the upper box. The numbers of γH2AX and 53BP1 foci in HeLa cells were quantified via immunofluorescence at late (A) and early (B) time-points after γ-irradiation. Where indicated, the cells were treated with KU55933, VE-822, or NU7441. Bars show means ± s.d. The numbers of cells counted in each condition are inserted in the graphs. Scale bars, 10 µm.

Next, we examined the involvement of the DNA damage checkpoint response in APOBEC3B activation after γ-irradiation. The kinases involved in this response, namely, ataxia-telangiectasia mutated (ATM), ATR, and DNA-PK, were inhibited by treatment of APOBEC3B-expressing cells with KU55933, VE-822, and NU7441, respectively (Fig. 2A). The late-onset γH2AX foci formed via APOBEC3B were efficiently induced in the presence of KU55933 or NU7441; however, their formation was specifically blocked in the presence of VE-822, suggesting a requirement for ATR. We also examined the effects of inhibiting ATR and ATM on early DNA damage responses (Fig. 2B). As expected, early-onset formation of γH2AX and 53BP1 foci after γ-irradiation was reduced in the presence of KU55933 (Fig. 2B), confirming the role of ATM in immediate responses to DSBs [19]. However, this had no effect on the frequency of late-onset DSB formation. Taken together, these results suggest that the deaminase APOBEC3B is activated by DSBs specifically via the ATR kinase. This results in C>U transitions that ultimately lead to accumulation of late-onset γH2AX-foci via UNG-mediated BER. Since ATR dependence is also observed during deamination-mediated mutagenesis under replication stress [20], ATR might be commonly involved in deamination.

### 3.3 APOBEC3B is stabilized in response to DSBs

**Fig. 3:**
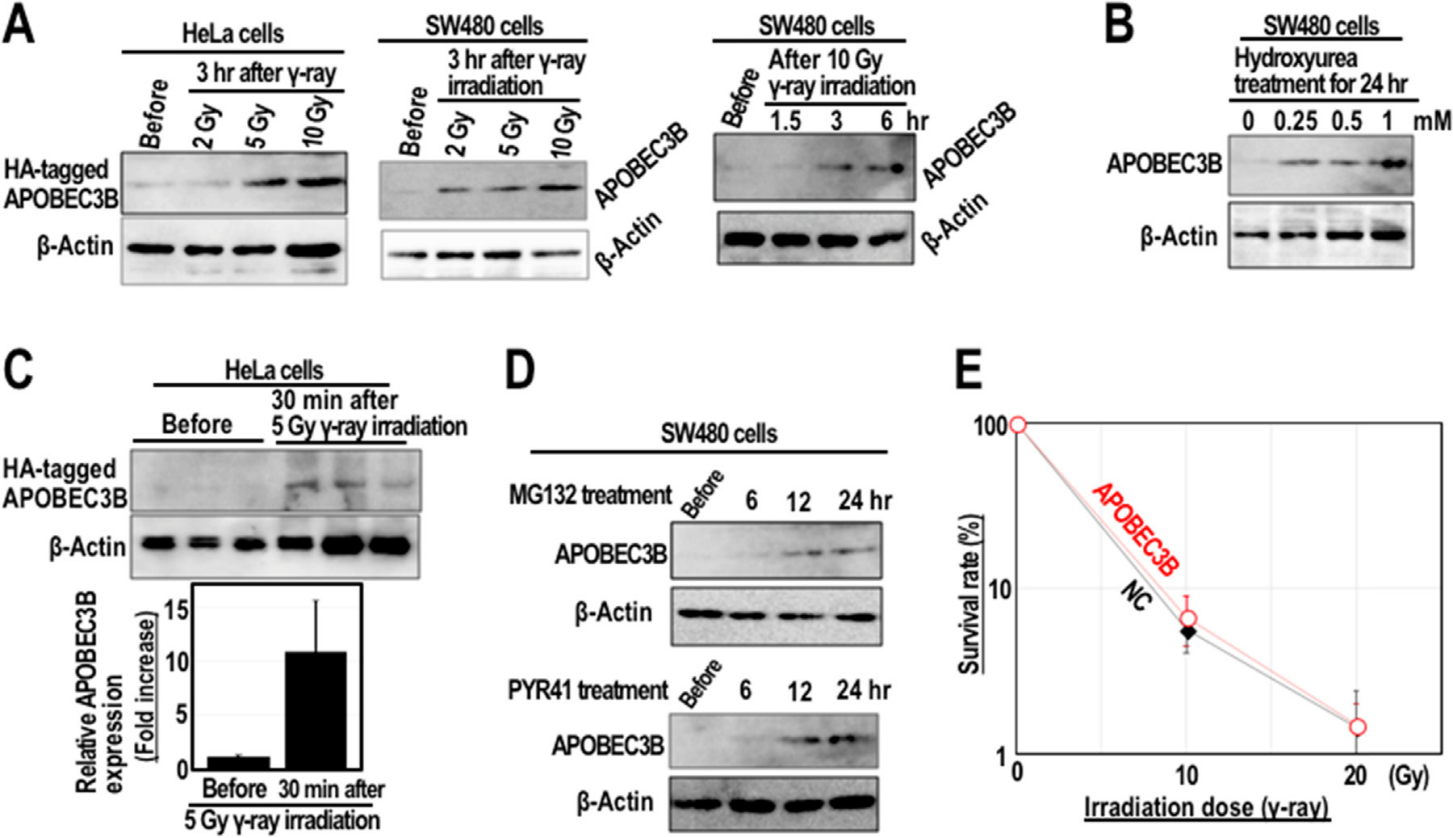
APOBEC3B is stabilized in response to DSBs. (A–C) HeLa and SW480 cells were irradiated by γ-ray (A, C) or treated with hydroxyurea (B). The expression of APOBEC3B and HA-tagged APOBEC3B was analyzed by western blotting. Western blotting was performed using an anti-HA antibody to detect exogenous HA-tagged APOBEC3B in HeLa cells and an anti-APOBEC3B antibody to detect endogenous APOBEC3B in SW480 cells. Bars show means ± s.d. (n = 3 biologically independent experiments). (D) SW480 cells were treated with MG132 or PYR41 as indicated. Western blotting was performed using an anti-APOBEC3B antibody. (E) HeLa cells expressing HA-tagged APOBEC3B and the negative control were irradiated by γ-ray. Their survival efficiencies were assessed. The graph shows mean survival rate (%) ± s.d. (n = 3 biologically independent experiments).

Deaminase-mediated mutations occur in association with kataegis (localized hypermutation at or around DNA rearrangement loci) [12], suggesting that deamination reaction is specifically induced at or around DSB sites that occur following DNA damage or during the repair process. To determine the damage dependence, APOBEC3B expression status was monitored after γ-irradiation, as APOBEC3B is usually not expressed in most cells and is specifically expressed in cells accumulated deamination-mediated mutations [21], [22]. As expected, the level of APOBEC3B protein was increased after γ-irradiation or treatment of hydroxyurea, an agent that causes replication stress-associated DSBs (Figs. 3A and 3B), indicating damage-dependent stabilization. The increase in APOBEC3B expression was observed as early as 30 min after γ-irradiation (Fig. 3C), which is much faster than the expression level changes that are usually seen following modulation of transcription and translation. One possible explanation is the involvement of proteasomal degradation, a process in which proteins are degraded under normal conditions. Such proteolysis is blocked in response to certain types of damage/stress, resulting in rapid accumulations of proteins, such as those observed for H2AX and NRF2 in response to DSBs and oxidative stress, respectively [23], [24]. To examine the hypothesis that inhibition of proteasomal degradation results in APOBEC3B accumulation, we treated SW480 cells with the proteasome inhibitor MG132 or the E1 ubiquitin ligase inhibitor PYR41. Remarkably, APOBEC3B was accumulated in non-irradiated cells following treatment with these agents (Fig. 3D). These results suggest that the deaminase APOBEC3B is continuously synthesized and is proteolyzed under normal conditions, but is immediately accumulated in response to DSBs via inhibition of the proteasomal-degradation pathway, ultimately causing deamination-mediated C>U transitions and subsequent long-lasting DSB formation.

To determine the effect of those deaminase-mediated secondary DSBs for radiation sensitivity, we examined the killing efficiencies of cells expressing APOBEC3B and compared with the control after radiation and observed almost no difference in those two conditions (Fig. 3E). This suggests that the effect of such late-onset DSB formation is probably limited at least for cancer-cell killing by IR.

## 4 Discussion

The results presented here demonstrate that the deaminase APOBEC3B is activated in response to DSBs, specifically through ATR kinase. In addition to causing mutagenic 5me-C>T transitions, APOBEC3B-mediated deamination reactions also cause C>U transitions, which subsequently induce UNG-mediated BER to eliminate uracil from the DNA. As a result, cells expressing APOBEC3B accumulate late-onset γH2AX foci as by-products of BER. Notably, the levels of late-onset γH2AX foci observed here were comparable to those of the primary γH2AX foci caused directly by γ-irradiation (Fig. 1, Fig. 2), suggesting massive BER induction.

### 4.1 DSB-triggered APOBEC3B stabilization

Deaminase-mediated mutagenesis is induced in association with kataegis, suggesting that deamination is very specifically induced at or around DSB sites. The precise mechanism by which APOBEC3B is induced is still unclear; however, the results presented here provide some insights. APOBEC3B is rapidly up-regulated in response to γ-irradiation. Such a rapid increase in expression is not possible through changes in transcription and translation rates, but can occur for factors that are continuously synthesized and degraded by the proteasome pathway. In fact, our experiments revealed that APOBEC3B is continuously synthesized and degraded by the proteasomal system under normal conditions. Notably, this type of rapid up-regulation of expression is usually seen during immediate responses to certain types of stress or damage; for example, H2AX and NRF2 are upregulated rapidly in response to DSBs and oxidative stress, respectively [23], [24]. Therefore, we suggest that APOBEC3B is regulated in a similar manner to enable immediate cellular responses to stress or damage.

Deaminases cause C>T/U transitions often in association with hypermutation at very limited loci, as seen in inflammation-associated cancer cells and in B cells during somatic hypermutation, specifically at variable regions of immunoglobulin genes [25], [26], [27]. Intriguingly, both are associated with DSB formation and genomic rearrangement, although the mechanism by which deamination-mediated mutagenesis is associated with DSBs is still elusive.

### 4.2 Long-lasting DSB formation

**Fig. 4:**
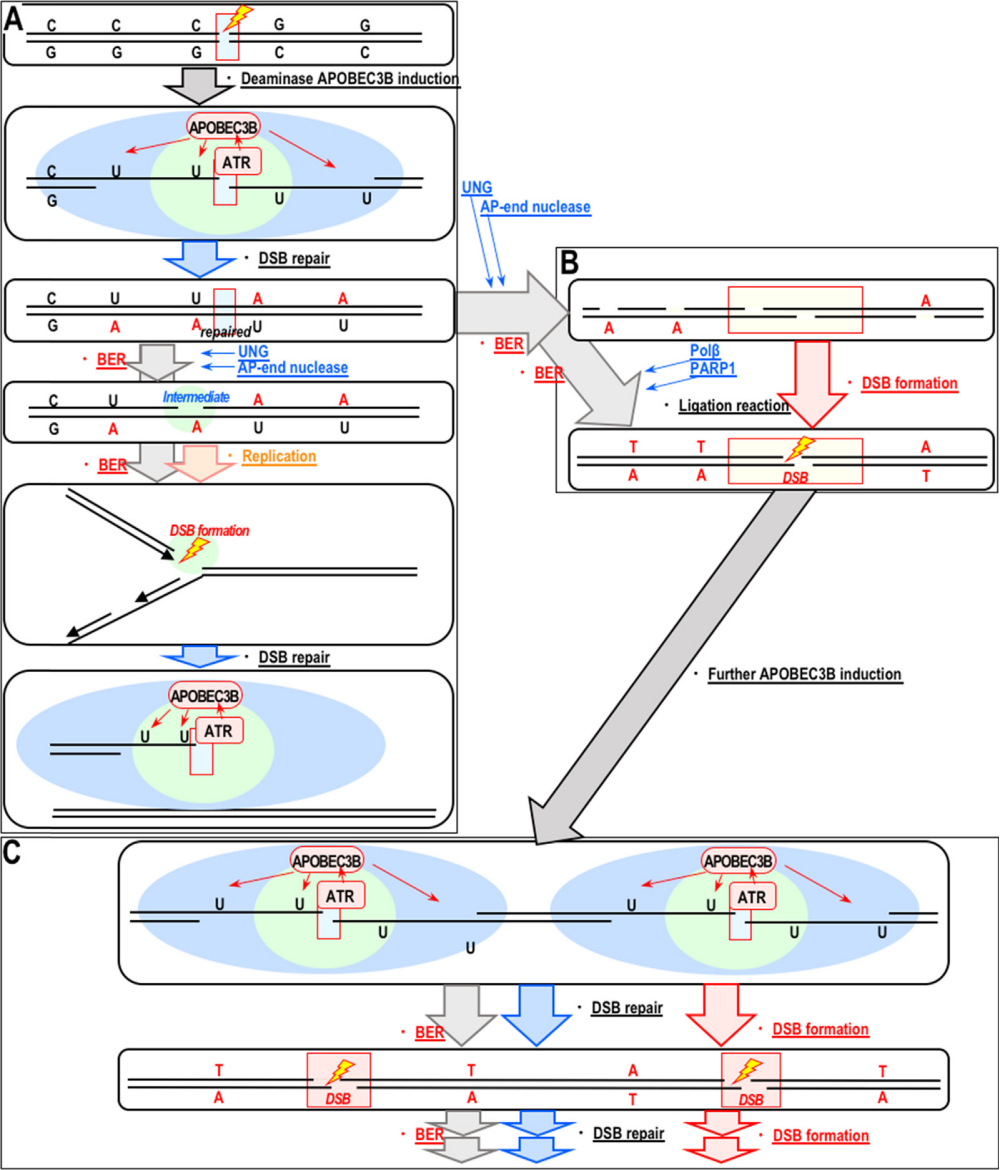
Proposed model. (A) APOBEC3B is activated in an ATR-dependent manner following γ-irradiation. The cells then undergo BER to eliminate uracil from the DNA, and some cells undergoing DNA replication can form DSBs. (B–C) Alternatively, massive deamination reactions are induced in very limited regions and cause further DSBs during the subsequent BER process (B). This event could lead to futile chain reactions of DSB formation and repair (C).

UNG-mediated late-onset γH2AX foci were continuously observed in most APOBEC3B-expressing cells 12–48 h after γ-irradiation (Fig. 1), indicating that such secondary DSBs are massively induced and difficult to repair. This finding is unexpected because BER-mediated DSB formation occurs during replication (or sometimes in association with transcription) and is therefore expected to occur only in some cells, specifically those in which DSBs are repairable (Fig. 4A). However, the frequency and persistence of the DSBs observed in the current study were much higher than expected. It is possible that secondary DSB formation is directly associated with the mechanisms of deamination and BER. In fact, since deamination-mediated hypermutation is induced in very limited regions at or around genome rearrangement loci (DSB sites) [12], deamination-mediated C>U transitions should be induced in very close proximity to these sites, at which spontaneous formation of more DSBs is expected as a consequence of BER (Fig. 4B). Such BER-mediated formation of DSBs could induce further deamination and cause further BER-mediated DSBs, resulting in multiple rounds of DSB formation and repair (Fig. 4C). It is not clear whether the late-onset DSBs observed herein undergo such futile cycles; however, this type of chain reaction could certainly contribute to their persistence.

It was recently shown that APOBEC3A/B expressing cancer cells are sensitive to ATR inhibitors owing to the resulting replication catastrophe [28]. These findings are likely different from our own because formation of late-onset long-lasting DSBs is due primarily to γ-irradiation and is blocked by an ATR inhibitor. Thus, the effects of ATR-mediated deamination reactions are likely context-dependent.

### 4.3 Effect of long-lasting DSBs caused by APOBEC3B

As shown in Fig. 3E, expression of APOBEC3B does not contribute to the killing effect of γ-irradiation. This might be due to the associated damage levels. In fact, when significant damage is caused by γ-irradiation, cells respond directly by inducing apoptosis via activation of damage checkpoints; therefore, in such cases, the effects of late-onset DSBs are probably limited. By contrast, APOBEC3B-mediated secondary DSBs arise only after repair of primary DSBs, in which damages caused by γ-irradiation must be tolerable for those cells. The number of secondary γH2AX foci is similar to that of primary foci; hence, the cells are expected to tolerate the damage. This may be why cells survive with long-lasting secondary DSBs.

APOBEC3B-mediated long-lasting DSBs could be a risk factor for genomic rearrangements. In fact, deamination-mediated hypermutation is observed in association with kataegis, which is a localized hypermutation at genome rearrangement loci [12]. This implies that the locus subjected to DSBs with massive deamination likely undergoes genomic rearrangement rather than direct end-joining repair. It is widely accepted that genomic rearrangements are caused by erroneous end-joining of DSBs, each of which is located originally at separated chromosomal positions. Such risks could increase when DSBs are not repaired effectively and/or when these DSBs occur in close proximity to other broken ends. Thus, formation of long-lasting DSBs likely increases the risk of genomic rearrangement.

## Funding

This study was supported by the MEXT Grants-in-Aid, Japan (20770136).

## References

[1] Lengauer C., KW K., Vogelstein B. (1997). Genetic instability in colorectal cancers. Nature.

[2] Lengauer C., Kinzler K.W., Vogelstein B. (1998). Genetic instabilities in human cancers. Nature.

[3] Nik-Zainal S., Wedge D.C., Alexandrov L.B., Petljak M., Butler A.P., Bolli N., Davies H.R., Knappskog S., Martin S., Papaemmanuil E., Ramakrishna M., Shlien A., Simonic I., Xue Y., Tyler-Smith C., Campbell P.J., Stratton M.R. (2014). Association of a germline copy number polymorphism of APOBEC3A and APOBEC3B with burden of putative APOBEC-dependent mutations in breast cancer. Nat. Genet..

[4] Chahwan R., Wontakal S.N., Roa S. (2010). Crosstalk between genetic and epigenetic information through cytosine deamination. Trends Genet..

[5] Shinohara M., Io K., Shindo K., Matsui M., Sakamoto T., Tada K., Kobayashi M., Kadowaki N., Takaori-Kondo A. (2012). APOBEC3B can impair genomic stability by inducing base substitutions in genomic DNA in human cells. Sci. Rep..

[6] Caval V., Suspène R., Vartanian J.-P., Wain-Hobson S. (2014). Orthologous mammalian APOBEC3A cytidine deaminases hypermutate nuclear DNA. Mol. Biol. Evol..

[7] Siriwardena S.U., Chen K., Bhagwat A.S. (2016). Functions and malfunctions of mammalian DNA-cytosine deaminases. Chem. Rev..

[8] Gallinari P., Jiricny J. (1996). A new class of uracil-DNA glycosylases related to human thymine-DNA glycosylase. Nature.

[9] Hendrich B., Hardeland U., Ng H.-H., Jiricny J., Bird A. (1999). The thymine glycosylase MBD4 can bind to the product of deamination at methylated CpG sites. Nature.

[10] Krokan H.E., Bjøra M. (2013). Base excision repair. Cold Spring Harb. Perspect. Biol..

[11] Blokzijl F., De Ligt J., Jager M., Sasselli V., Roerink S., Sasaki N., Huch M., Boymans S., Kuijk E., Prins P., Nijman I.J., Martincorena I., Mokry M., Wiegerinck C.L., Middendorp S., Sato T., Schwank G., Nieuwenhuis E.E.S., Verstegen M.M.A., Van Der Laan L.J.W., De Jonge J., Ijzermans J.N.M., Vries R.G., Van De Wetering M., Stratton M.R., Clevers H., Cuppen E., Van Boxtel R. (2016). Tissue-specific mutation accumulation in human adult stem cells during life. Nature.

[12] Nik-Zainal S., Alexandrov L.B., Wedge D.C., Van Loo P., Greenman C.D., Raine K., Jones D., Hinton J., Marshall J., Stebbings L.A., Menzies A., Martin S., Leung K., Chen L., Leroy C., Ramakrishna M., Rance R., Lau K.W., Mudie L.J., Varela I., McBride D.J., Bignell G.R., Cooke S.L., Shlien A., Gamble J., Whitmore I., Maddison M., Tarpey P.S., Davies H.R., Papaemmanuil E., Stephens P.J., McLaren S., Butler A.P., Teague J.W., Jönsson G., Garber J.E., Silver D., Miron P., Fatima A., Boyault S., Langerod A., Tutt A., Martens J.W.M., Aparicio S.A.J.R., Borg Å., Salomon A.V., Thomas G., Borresen-Dale A.L., Richardson A.L., Neuberger M.S., Futreal P.A., Campbell P.J., Stratton M.R. (2012). Mutational processes molding the genomes of 21 breast cancers. Cell.

[13] Hoopes J.I., Cortez L.M., Mertz T.M., Malc E.P., Mieczkowski P.A., Roberts S.A. (2016). APOBEC3A and APOBEC3B preferentially deaminate the lagging strand template during DNA replication. Cell Rep..

[14] Minakawa Y., Atsumi Y., Shinohara A., Murakami Y., Yoshioka K. (2016). Gamma-irradiated quiescent cells repair directly induced double-strand breaks but accumulate persistent double-strand breaks during subsequent DNA replication. Genes Cells.

[15] Ichijima Y., Yoshioka K., Yoshioka Y., Shinohe K., Fujimori H., Unno J., Takagi M., Goto H., Inagaki M., Mizutani S., Teraoka H. (2010). DNA lesions induced by replication stress trigger mitotic aberration and tetraploidy development. PLoS One.

[16] Atsumi Y., Fujimori H., Fukuda H., Inase A., Shinohe K., Yoshioka Y., Shikanai M., Ichijima Y., Unno J., Mizutani S., Tsuchiya N., Hippo Y., Nakagama H., Masutani M., Teraoka H., Yoshioka K. (2011). Onset of quiescence following p53 mediated down-regulation of H2AX in normal cells. PLoS One.

[17] Refsland E.W., Harris R.S. (2013). The APOBEC3 family of retroelement restriction factors. Curr. Top. Microbiol. Immunol..

[18] Ensminger M., Iloff L., Ebel C., Nikolova T., Kaina B., Löbrich M. (2014). DNA breaks and chromosomal aberrations arise when replication meets base excision repair. J. Cell Biol..

[19] Shiloh Y., Ziv Y. (2013). The ATM protein kinase: regulating the cellular response to genotoxic stress, and more. Nat. Rev. Mol. Cell Biol..

[20] Kanu N., Cerone M.A., Goh G., Zalmas L.-P.L., Bartkova J., Dietzen M., McGranahan N., Rogers R., Law E.K., Gromova I., Kschischo M., Walton M.I., Rossanese O.W., Bartek J., Harris R.S., Venkatesan S., Swanton C. (2016). DNA replication stress mediates APOBEC3 family mutagenesis in breast cancer. Genome Biol..

[21] Burns M.B., Lackey L., Carpenter M.A., Rathore A., Land A.M., Leonard B., Refsland E.W., Kotandeniya D., Tretyakova N., Nikas J.B., Yee D., Temiz N.A., Donohue D.E., McDougle R.M., Brown W.L., Law E.K., Harris R.S. (2013). APOBEC3B is an enzymatic source of mutation in breast cancer. Nature.

[22] Tsuboi M., Yamane A., Horiguchi J., Yokobori T., Kawabata-Iwakawa R., Yoshiyama S., Rokudai S., Odawara H., Tokiniwa H., Oyama T., Takeyoshi I., Nishiyama M. (2016). APOBEC3B high expression status is associated with aggressive phenotype in Japanese breast cancers. Breast Cancer.

[23] Atsumi Y., Minakawa Y., Ono M., Dobashi S., Shinohe K., Shinohara A., Takeda S., Takagi M., Takamatsu N., Nakagama H., Teraoka H., Yoshioka K. (2015). ATM and SIRT6/SNF2H mediate transient H2AX stabilization when DSBs form by blocking HUWE1 to allow efficient γH2AX foci formation. Cell Rep..

[24] Kobayashi A., Kang M., Okawa H., Ohtsuji M., Zenke Y., Chiba T., Igarashi K., Yamamoto M. (2004). Oxidative stress sensor Keap1 functions as an adaptor for Cul3-based E3 ligase to regulate proteasomal degradation of Nrf2. Mol. Cell. Biol..

[25] Takai A., Marusawa H., Chiba T. (2011). Acquisition of genetic aberrations by activation-induced cytidine deaminase (AID) during inflammation-associated carcinogenesis. Cancers (Basel).

[26] Mechtcheriakova D., Svoboda M., Meshcheryakova A., Jensen-Jarolim E. (2012). Activation-induced cytidine deaminase (AID) linking immunity, chronic inflammation, and cancer. Cancer Immunol. Immunother..

[27] Chiba T., Marusawa H., Ushijima T. (2012). Inflammation-associated cancer development in digestive organs: mechanisms and roles for genetic and epigenetic modulation. Ygast.

[28] Buisson R., Lawrence M.S., Benes C.H., Zou L. (2017). APOBEC3A and APOBEC3B activities render cancer cells susceptible to ATR inhibition. Cancer Res..

